# Grey Zone Healers and the COVID-19 Pandemic in Chechnya, Russia

**DOI:** 10.1007/s10943-024-02041-4

**Published:** 2024-04-09

**Authors:** Evgenia Zakharova, Iwa Kołodziejska, Iwona Kaliszewska

**Affiliations:** Faculty of Culture and Arts, Institute of Ethnology and Cultural Anthropology, University of Warsaw, ul. Żurawia 4, 00-503 Warsaw, Poland

**Keywords:** CAM healers, Tactics, COVID-19, Chechnya, North Caucasus

## Abstract

The Chechen authorities’ focus upon population health is enacted both through the principles of Islamic medicine and approved biomedical practices. Any healing practices beyond these domains are met with deep suspicion. Practitioners of unofficial complementary and alternative medicine healers may abruptly find themselves regarded as enemies of the state. In light of this precarious circumstance, it becomes pertinent to inquire: How do these healers employ their daily tactics to negotiate the intricate power dynamics between the formidable state apparatus and the established biomedical order? Drawing from our meticulous fieldwork conducted in the year 2021, we investigated the intricate tactics employed by unofficial healers in the Chechen medical landscape during COVID-19. Our research centred on discerning the nuanced tactics aimed at mitigating potential risks. We conclude that healers, having embodied tactics to creatively manoeuvre within the confines of the authoritarian state, perceived the challenges posed by COVID-19 as merely another, often inconsequential, obstacle in their enduring struggle.

## Introduction

In Chechnya, a constituent republic in the North Caucasus, assuming the mantle of an unofficial complementary or alternative medicine healer is an enterprise fraught with risk and uncertainty. The Chechen authorities’ focus on the population’s health is enacted through a combination of Islamic medicine and official biomedical practices. Any healing practices that venture beyond these domains are met with suspicion, thereby rendering unconventional therapeutic roles perilous within this framework. Doctors, akin to their counterparts throughout the Russian Federation, operate within the parameters of a biomedical system, while “official” healers are institutionalized in the state-funded Islamic Medical Centre established in 2009, where patients undergo healing procedures in accordance with the principles of traditional Islamic medicine.

An “unofficial” healer may, overnight, become an enemy of the state, or an enemy of the state-orchestrated understanding of Islam. With a state-promoted tradition of collective responsibility, the danger of repercussions extends beyond the individual in question, enveloping also their family. The only way to dispel the allegation of sorcery (which may result in detention) is to publicly renounce one’s involvement in “witchcraft”, followed by a humble apology broadcasted by the Chechen state television, effectively equivalent to public shaming. Remarkably, the “unofficial” healers in Chechnya showed great perseverance, both before and during the COVID-19 pandemic.

Within the Western context, violence in medical practices is often associated with the hegemony of official medicine. Healing practices inspired by religion are often viewed with disdain in the Western context, and are often pushed into the margins of the medical discourse; sometimes even into the grey zone (Roussou, 2021).^1^ The state medical system and practitioners of biomedicine typically either reject or, at best, exhibit mild acceptance or reluctant approval for what is commonly referred to as “complementary medicine” (e.g. Germany–Frank & Stollberg, 2006; Portugal and Greece–Roussou, 2021).

In contrast, religious healing in Chechnya takes a central position within the official medical landscape, standing shoulder to shoulder with biomedicine. Religious healing is not only endorsed, but promoted as traditional and effective. Conversely, the healing practices diverging from the approved framework are deemed suspicious and relegated to the ambiguous “grey zone”. It is precisely this territory that we want to explore further in this paper, trying to understand the tactics employed by the healers in order to navigate professional and societal existence in this particular context.

In this paper, we will introduce the term “grey zone healers” and analyse the tactics (sensu de Certeau, 1984) employed by practitioners of Complementary and Alternative Medicine (CAM) in Chechnya, who operate on the precipice of legality, risking possible harassment from authorities. The grey zone healers function within a spectrum of legality that encompasses both sanctioned and semi-sanctioned practices. This categorisation does not reflect mere financial considerations, but instead the delicate balance between actions deemed acceptable or otherwise by the authorities—a situation often further complicated by often unclear directives of the Chechen president.

We will focus on healers who apply methods primarily focused on the corporeal realm, that is the body itself. In this approach, the healing process starts with the patient’s body rather than their spiritual state. The healers who focus primarily on the latter, for example through referring to clairvoyance, or finding the source of an “evil eye”, are subject to heightened state surveillance and potential violence. Although these healing processes begin with, or are rooted in, bodily manipulations, the understanding of health is decidedly holistic. The healers address not only the physical ailment, but also the broader spectrum of sickness, including its social and spiritual dimension.

We understand their practices as complementary medicine therapeutic methods, because they do not alienate themselves completely from biomedicine; instead, they see and often advocate for the prospect of integrating it within their healing practice.^2^ Our research was conducted during the period of the Sars-Cov-2 pandemic, an event globally perceived as a catalyst for intensified state surveillance and violence. Consequently, understanding the situation of grey zone healers, individuals already acclimated to functioning with prevalent presence of state violence, became a subject of our profound interest within this particular context. In our research group, the healers employed diverse treatments, such as chiropractic, herbal medicine, apitherapy (honeybee products therapy), hirudotherapy (medicinal leech therapy), or homeopathy. They adeptly incorporated the discourse of the pandemic, presenting their methodologies as not only applicable, but also effective for the virus treatment.^3^ They had to address the SARS-CoV-2 virus itself, as well as navigate the social and state response to it.

Fourteen years of state-orchestrated persecutions contributed to the formation of grey zone healing practices, which became deeply embedded into the medical landscape of Chechnya. The concept of “medical landscape” is useful here because, as Hsu points out «[r]ather than invoking a clearly bounded culture concept with a culturally adept healer in its centre, the notion of medical landscapes implies social processes, relatedness, and movements between foregrounds and backgrounds, and across boundaries» (Hsu, 2008, p. 320). This concept allows us to discern the dynamic and fluid nature of social reality, including the intricate relations and actions of its participants. Medical landscapes exhibit distinctly individual hierarchies varying from person to person. Each grey zone healer constructs a unique medical landscape of their own, shaped by their personal interests and resources.

In this paper, we delve into the intricacies of how grey zone healers navigate the medical landscape during COVID-19, how they narrate themselves, and what tactics (sensu de Certeau, 1984) they employ to evade possible harassment from authorities. How do they navigate between the hegemonies of the state apparatus and biomedicine? What is their relationship with official Islamic medicine in Chechnya?

We will show that state-driven persecutions are reducing healers’ medical landscapes’ diversity. Within these landscapes, the healers act in accordance with the relations they have with various entities (other people, institutions, medicines, plants, and animals they use in the healing process). In Chechnya, such healers are compelled to comply, or at least simulate compliance with, the state-sanctioned Islamic medicine and the narratives of the Chechen national identity. Even prior to the COVID-19 pandemic, healers in Chechnya skilfully manoeuvred in their medical landscapes, and adapted to unfavourable conditions. The emergence of SARS-CoV-2, as we show, was for them just another obstacle, layered upon the existing obstacles posed by the authoritarian state. Nevertheless, it was an obstacle they could not disregard, and they had to employ effective tactics to mitigate the novel risks precipitated by the state.

In 2006, Marcia Inhorn and Carolyn Sargent highlighted a significant gap in medical anthropology research and literature within Islamic contexts (Inhorn & Sargent, 2006). In recent years, issues relating to health and religion within Muslim communities composed the most prominent single cluster in the bibliometric survey of the *Journal of Religion and Health*. These articles, however, predominantly focused on the influence of Islamic beliefs upon the health of American Muslims (Carey et al., 2023), with none focused on the Muslims in the post-Soviet region.^4^ Islam in this area possesses unique characteristics, prompting us to contend that research from Chechnya, a republic inaccessible to most researchers, holds the potential to enrich not only the Islamic or the post-Soviet area of studies, but it could also contribute to the broader field of medical anthropology literature.^5^

## Data and Methods

Conducting fieldwork in the North Caucasus, particularly in Chechnya, is severely restricted. Researchers must exercise extreme caution, prioritising the safety of both their informants and themselves. Avoiding interactions with individuals persecuted by the authorities (including some healers) is therefore strongly advised. Consequently, while one of the authors had engaged with such individuals in 2009/10, the more recent fieldwork in 2021 and 2022 (designed collectively but conducted by Author1) mandated a cautious approach. Due to safety concerns, it was decided not to interview these vulnerable people.

The fieldwork was conducted during two field seasons: one month spanning August to September 2021, in the city of Grozny and several villages of the Chechen Republic, followed by two months in January to February and March to April 2022, in Grozny.^6^ Stringent security measures were implemented to safeguard the informants. Not all interviews were recorded, and particular questions deemed overly sensitive were judiciously omitted.

The article is predominantly based on semi-structured interviews with nine healers, aged from 35 to 80 years. Among them were: an apitherapist, two chiropractors, three herbalists, and three naturopaths—a hirudotherapist, an acupuncturist, and a practitioner of the Bolotov Method.^7^ Three of these individuals operate their own medical clinics, with one being a family-run establishment, while the other two employ staff members from outside the founders’ familial circles (cf. Table 1).

**Table 1:** Key participants’ demographic characteristics

Name	Gender	Age	Practice type
Elmira	Female	~ 35	Naturopath–hirudotherapist
Hasan	Male	~ 60	Chiropractor, founder of the multidisciplinary clinic
Loma	Male	~ 67	Apitherapist, has apitherapy clinic, plans to organize multidisciplinary clinic
Sultanbek	Male	~ 84	Naturopath–Bolotov follower
Sultan	Male	~ 48	Chiropractor
Vezirkhan	Male	~ 62	Herbalist
Yahita	Female	~ 65	Herbalist
Yusup	Male	~ 60	Herbalist
Zakir	Male	~ 47	Naturopath–acupuncturist, owner of a complementary medicine clinic, biomedical doctor

All names have been changed

We conducted 26 interviews with healers, doctors (working within both private and as well as state-run hospitals and clinics), and their patients. Among the interviewed healers, five resided in large suburban villages near Grozny, two were inhabitants of Grozny city, and the remaining two lived at a distance from the capital. Supplementary material for this study was derived from field notes on participant observation in hospitals, clinics, polyclinics, pharmacies, or Islamic stores, conducted in 2021/22. Author 3’s material from 2009/2010 provided additional data on state violence.

This article will apply the term “healers” to refer to all discussed complementary medicine specialists. This designation is *etic* in nature, as none of the interlocutors used this term to self-reference.

As a precautionary measure, all healers were pseudonymized to safeguard their identities. Although this level of protection might not have been strictly necessary in all instances, we opted to err on the side of caution, to ensure that no personal and potentially harmful data would be disclosed. The study was performed in line with the Principles of Professional Responsibility of AAA (PPR, 2012). The ethical commission of the Institute of Ethnology and Cultural Anthropology at the University of Warsaw reviewed and approved the study methodology.

### Chechnya: State, Islam and State-Supported Islamic Medicine

Chechnya is a small republic in the North Caucasus region of the Russian Federation. Its population counts approximately 1.3 million people and is predominantly concentrated in and around the capital city of Grozny. Despite the ostensible normalcy when perceived from an external perspective, the indelible mark of two devastating recent wars is clearly visible. Similar to its sister republics on the surface, upon closer inspection Chechnya reveals a landscape rife with insecurities, pervasive fears, and profound mistrust towards the authorities and fellow citizens.

The republic enjoys a special status and significant political autonomy within the Russian Federation. Under the leadership of Ramzan Kadyrov, Chechnya turned into a region of "personal subordination" to both Kadyrov and Vladimir Putin (Milashenko, 2019), thus gaining a semblance of independence, albeit within the strict confines of the Russian State. The government of Chechnya assumed an autocratic nature, taking traditionalism and Sufi Islam as its ideological basis (Raubisko, 2009), with the aim to strictly regulate the religious life of Chechens, extending to the minutiae of dictating sermon topics for local imams.

The strain of Islam propagated by the authorities derives from the practices of the Qadiriyya Sufi brotherhood,^8^ and is established as an opposition to Salafi Islam. In consequence, the Salafis are persecuted in the republic and their existence is officially denied (Raubisko, 2009). This results in a conspicuous absence of a public discourse on religious practices other than those officially acknowledged. In effect, Chechnya manifests as a totalitarian, quasi-state entity that strongly intervenes in the lives of its citizens, extending not only to their religious practices, but also health, healing practices, and their general well-being.

As an integral part of the Russian Federation, Chechnya provides its residents with free-of-charge access to medical services, acknowledged for their reputed decent quality. Patients availing these services were, however, very critical of the state medical system, alleging pervasive corruption and bribery for various treatments. The COVID-19 pandemic, according to their accounts, brought no changes in this regard. The patients were burdened with medication expenses and frequently, unnecessary additional blood tests that the doctors recommended. These corruption practices and the ensuing discourses are also present in the neighbouring republics (Kaliszewska & Schmidt, 2022), and as such constitute an important part of the broader medical landscape of the region.

In Chechnya, as in other regions of the North Caucasus, the phenomenon of Islamic medicine^9^ experienced a significant proliferation in recent years. Islamic medicine represents a diverse set of remedies and methods associated with Islam and the Quran. Essentially constituting complementary medicine, its legitimacy is derived from the Islamic beliefs. In Chechnya Islamic medicine has received unequivocal endorsement from the state. The remedies and medicines linked to Islam and the Quran are widely perceived as effective and broadly applicable. Notably, during the COVID-19 pandemic, remedies such as qust/costus (*Sassurea lappa*), or black cumin (*Nigella sativa*) oil, predominantly imported from Gulf countries, were widely advertised and used by the populace as preventive measures and agents against the novel virus.

While Islamic medicine, along with products imported from Gulf states or Egypt, partially links Chechnya to the broader umma,^10^ the “Chechenisation” of Islam and persecution of individuals who studied in the Middle East understandably curtail the unrestricted exchange of ideas, which need to pass through censorship. The scope of what constitutes Islamic medicine in Chechnya, and how it is applied, is defined both globally and locally, depending upon the current political agenda. Effectively, the only permitted form of Islamic medicine is the one officially certified and controlled by the state.

### Witch-Hunt. How do Grey Zone Healers Deal with State Violence?

Non-Islamic complementary medicine, and particularly the segment of it associated with traditional healing, became a target of intense scrutiny by Chechen authorities, and the practices broadly categorised as “magic” were subject to harsh punitive measures. The authorities’ understanding of terms such as “magic” or “sorcery” is arbitrary. The word “magic” encompasses practices such as fortunetelling, spellcasting, and practising black magic (e.g. burying photographies, or placing headless birds in graves). Practitioners of these activities were labelled as sorcerers (russ. *kolduny*), clairvoyants (*yasnovidcy, yasnovidyashche*), witch-doctors (*znakhary*), black wizards (*chiornye magi*), or healers (*celitieli*) (cf. Lindquist on magic and healing in Moscow in the 90 s’ (2006)). To define “magic” and “sorcerers”, the Chechen authorities focused on, magical paraphernalia, such as cards, sheets of paper with spells, bones, soil collected from cemeteries, photos of clients, and many others.

Suspicions towards various CAM healers in Chechnya first emerged in 2008, when Chechen authorities, in cooperation with the *muftiyat*,^11^ declared a “fight with sorcerers”.^12^ At the same time, the Chechen president presented the idea of the Islamic Centre, envisioned as a facility where patients would receive treatment rooted in Islamic medicine, particularly through the recitation of *ayat* from the Quran.^13^ "The clinic's focus is on unconventional methods of treating patients suffering from psycho-neurological disorders, locally recognised as possession", Kadyrov announced at the centre’s opening ceremony in 2009 (Groznom otkryli, 2009). The Islamic medicine practitioners gathered in the centre mastered Quranic recitation and Islamic healing, either through independent study, or in mosques; others, already engaged in their private healing practices, faced a choice of either ceasing their practice or integrating into the Islamic centre.

For instance, Adam, a healer the third Author talked to in 2010, practised clairvoyance and healing before the widespread persecutions. In 2010, the authorities instructed him to cease his practice, offering him an opportunity to reorient his healing methods and join the Islamic centre. Adam declined, and declared his practice closed, but he continued to operate on a smaller scale, catering to a select circle of trusted individuals. Within this “grey zone”, his focus shifted towards the physical well-being of his clients. Spiritual Islamic healing, according to *muftiyat*, was important due to the prevalence of psychological challenges experienced by many people in Chechnya—challenges purportedly beyond the scope of conventional medical approaches.

Chechnya’s state-operated Islamic Medical Centre was inaugurated in 2009 and soon boasted to have served 150 people daily for free by 2011, with numbers steadily increasing over time.^14^ Depression, often re-diagnosed as possession, proved to be the prevailing reason for visits (cf. Nguyen et al., 2016). In the subsequent years, allegations of sorcery gradually turned into a state-orchestrated witch-hunt. According to reports from *Nastoiashchee Vremja* (Current Time) TV, in February 2013, Kadyrov, upon discovering that a Chechen official (Ahmad Abasstov) sought the services of a wizard (*mag*) to bewitch (*zakoldovat’*) Kadyrov, compelling him to grant the official a ministerial position,^15^ Kadyrov demanded eradication of “such people” (witch-doctors, healers, fortune-tellers) in Chechnya. In a meeting with municipal leaders and *qadis* (Islamic judges), he publicly threatened them with police. As a result, a vigorous campaign was initiated and persecutions and abductions followed. The *muftiyat* justified these measures on the grounds of the contradiction between these practices and Islam. The “witch-hunt” extended beyond Chechnya, with healers targeted also in the neighbouring Dagestan, Ingushetia, and Kabardino-Balkaria.^16^

However, Chechnya stood unique with the state authorities orchestrating the persecutions—in other republics, individuals subscribing to a strict, puritan religious interpretation undertook responsibility, while the state was not involved.^17^ Islamic teachings consider practising magic a major sin, inviting severe retribution in the afterlife. In Chechnya, state authorities acted akin to the above-mentioned unofficial armed groups. Unlike these groups, the authorities were not driven by a rigid adherence to a normative Quranic understanding,^18^ but rather by their desire to exercise control and discipline.

To remain within the framework of Russian law, the healers were persecuted under the charge of fraud (*moshennichestvo,* cт.159 УК PФ), allegedly promising a treatment that would cure the patient’s illness, whereas offering remedies unlikely to deliver the desired result (e.g. an amulet). These two justifications, the “Islamic” one and the “rational” one, were most often evoked. Interestingly, while the authorities emphatically accused healers of fraud, patients attending the state-run facilities accused medical personnel of identical malpractice.

In 2019, further incidents of persecution occurred, accompanied by an element of public humiliation: those accused of witchcraft were coerced into confessing and apologising on state television, at the same time being reprimanded by Adam El’zhurkayev, the director of the Islamic Medical Centre. Over time, the authorities extended the witch-hunt to include clients of the alleged “sorcerers”, accusing them of planning to inflict harm on others by casting a spell on them.

In Chechnya, the act of publicly shaming an individual extends beyond the person and implicates their entire social network, especially their relatives. Chechnya upholds a widely acknowledged notion of collective responsibility. Consequently, humiliating an individual may threaten or anger their extended family, particularly its male members,^19^ who are expected to oversee younger family members—especially women. They may react in a variety of ways: either by sympathizing with or, conversely, critically denouncing the healer. Similarly “disgraced” women may face denouncement, or even death in the so-called honour-killings,^20^ carried out by their own relatives. This shaming practice in Chechnya evokes the tradition of collective responsibility, religious principles (a set of regulations one must adhere to), and remnants of the Soviet tradition, including the coercive practice of *samokritika*, or self-criticism.^21^

More recent detainments of “witches” resulted in their enforced “curing”, for instance, in November 2021, three women were arrested for practising witchcraft and fraud. To substantiate their alleged involvement, Adam Elzhurkayev, the head of the Islamic centre, declared that they were possessed by jinns. Following their public, broadcasted humiliation, Elzhurkayev asserted his ability to “cleanse” them through Quranic verses, application of oils, smoke inhalations, and the palms of his hands. Subsequently, the women were coerced into signing an agreement renouncing their practices. Their belongings associated with “sorcery” were confiscated, and the women were eventually released into the custody of their male relatives, who were now tasked with monitoring them.^22^ While the above-mentioned accusations lack logical coherence, not all healers were equally vulnerable. Based on our research, healers focusing on the “mind” (e.g. clairvoyants) were targeted more frequently than those dealing exclusively with physical ailments. This study concentrates on the latter group.^23^

Overall, the witch-hunt constituted a segment of wider repressive actions orchestrated within the Chechen republic, where “problematic” individuals were targeted because of their religious identity (such as Salafi or Wahhabi Islam^24^), their purported healing practices, or their sexual orientation.^25^ These “regulative” and disciplining measures persisted throughout our fieldwork and, based on our online interactions with the informants, they did not cease in the past year.

What tactics do grey zone healers employ when dealing with state violence? How does state violence influence their movement within medical landscapes during the COVID-19 pandemic? How do these healers respond to state-driven Islamization and re-traditionalization in the medical landscape?

During our research, we observed various tactics used by grey zone healers to deal with oppressive policies of state authorities. In de Certeau’s framework, tactics are in opposition to strategies, which are always set within the purview of power. Tactics represent adaptations to the environment, created by the strategies of those in power (1984). A healer may employ more than one tactic, and the conscious awareness of this choice can vary. This means that some healers may perceive certain tactics as ways of evading state oppression, but others do not view them in this way—even if the researchers do. State violence, although undeniable, is seldom openly discussed. Living and working in Chechnya, the healers adapted to the conditions in the republic, navigating its medical landscape with varying degrees of awareness and intent. In the following sections, we delve into the most prevalent tactics utilised by grey zone healers in Chechnya.

### Discursive Tactic: “I’m What the State Allows Me to Be”

To legitimize themselves in the eyes of patients as well as authorities, the grey zone healers stress their alignment with state-endorsed biomedicine. They highlight instances of medical doctors either using their remedies or prescribing them to their patients. This approach is used by Yusup, a herbalist in his 60s, whose expertise includes collecting medicinal herbs and preparing concoctions, teas, and other herbal products prescribed for an array of ailments. According to his narrative, his nephew, a biomedical doctor, recommends his teas to post-COVID patients: Yusup explains, “He [the doctor] knows that I have these types of teas. All these COVID, post-COVID patients are saying: ‘I can’t sleep’, ‘I’m constantly afraid’. In addition to the medicines that he prescribes, he also says ‘’go to my uncle’. And he takes my teas, I usually give him 100–150 packs.”

Elmira, a hirudotherapy practitioner in her late 30 s, operates a private practice using medicinal leeches. She explained that the prices for leeches increased significantly during the first wave of the pandemic, as private medical clinics and even public hospitals started utilising them. She attributes the leeches' efficacy in treating COVID-19 to their ability to thin the blood and substitute anticoagulants.^26^ In her view, “COVID-19 triggers the same inflammatory process in the lungs as pneumonia, and pneumonia is treated with leeches because there is an inflammation in the body.” She stresses the distinction between her approach and the biomedical methods, reaching for the “naturalness” argument, and expressing the conventional cliché about biomedical methods: “Using leeches is naturally better than injecting anticoagulants because medical intervention treats one thing, but harms the other.”

Loma, an apitherapist in his late 60s, discursively refers to biomedicine to demonstrate that his practice falls within the “accepted by the state” category. At the same time, he endeavours to distinguish himself from biomedicine, presenting it as less trustworthy than his own method: “People are not seeking treatment in biomedical healthcare facilities until the last moment, they have already lost confidence in them.” He does not discredit biomedicine as such, but critiques the inefficient system that fails to function effectively in Chechnya. His selective reference to biomedicine may thus be perceived as a tactic to narrate himself within the boundaries of the “permitted”^27^ and to be viewed and valued as a professional.

All interviewed healers distanced themselves from the label of “witchcraft” or “sorcery” (russ. *koldovstvo*)—a term used by the state authorities towards the healers they persecute. The analysis of the healers’ narratives reveals that, through nuanced rhetoric, they endeavour to emphasise their alignment with the state’s perspective. They claim to accept the state’s narrative about “witchcraft”, support the persecutions, and emphasise their distinction from the alleged “sorcerers”. For instance, Yusup strongly condemns such individuals, stating: "Witchcraft itself is punished, and punished very severely; it is very harmful, it is forbidden in Islam. But phytotherapy and witchcraft are [two] completely different things".

From the legal perspective, healers labelled as “*kolduny*” (sorcerers) face accusations of fraud (as witchcraft is not a penalized offence on its own). In consequence, all grey zone healers emphasise that they provide their services free of charge. This way they distance themselves from any semblance to sorcerers. Such narratives can be seen as a tactic that aligns them with practices compliant with the state’s narratives, potentially ensuring their safety.

The prohibition on the provision of commercial medical services without proper registration compels healers to present their occupation not as a medical practice, but as gratuitous assistance to those in need. This “gratuity” usually involves voluntary payments of an unspecified but well known amount, facilitated through various channels. The emphasis is placed on the services being offered free of charge, coupled with a narrative of “helping” (“helping over curing”). Yusup elucidates: “… it would be probably wrong to say that I'm treating COVID. People know that I work with herbs, they know that I have all sorts of lung herbs, so they come to me. I say:’I don’t know, it helped me, if you want you can take it…’. I don’t treat people, I don’t take money,^28^ I earn money with my tea, and I also make healing ointments. I earn money this way, but pneumonia, tuberculosis, all these diseases, if I can help, if I can offer something, I offer it to people and explain to them how to use it and where to get it from. So, it does not work this way, that a person comes to me with a specific disease and I treat them.”

Providing assistance without the expectation of monetary compensation (even though payments are often accepted in the form of gratuity) distinguishes the grey zone healers' medical practices from the broader biomedical landscape, where unofficial payments are commonplace.^29^ To ensure safety, or simply to diversify their family’ income, some grey zone healers pursue other sources of revenue. For instance, Elmira is working as an event manager, while Sultan, a chiropractor in his late 40 s, tends to a family farm and sells self-produced flour. Loma openly advertises and sells his honey products and participates as an exhibitor in international beekeeping and honey shows.

As individuals holding diverse professional roles, grey zone healers avoid being directly associated with financial gains from their healing practices. Alternative employment may also serve as a cover for their grey zone healing practice.^30^ Having another employment, or switching from a different job to CAM activities is a commonplace practice among healers across different cultural contexts (e.g. Sharma, 1992),^31^ but in the confines of a violent authoritarian state, it serves the above-mentioned auxiliary function.

During the interviews, the healers tended to avoid explicitly mentioning COVID-19 in the context of their healing practices, although they did treat patients suffering from COVID-19 and post-COVID symptoms. With the exception of Loma, an apitherapy practitioner, none of the individuals initiated conversations with the researcher about COVID-19 or the treatments they could offer for the disease; in no case was COVID treatment presented as their calling card. Yusup, as cited above, when asked directly about COVID-19, stated that “it would be probably wrong to say that I'm treating COVID”. In his narration, the responsibility for the use of his herbal teas in treating COVID-19 was given to his nephew, a biomedical doctor.

Loma, who advertised his treatment with propolis (a resin created by bees) on Instagram in April 2020, praised its effectiveness for “virus-caused pneumonia and various virus diseases”, but he never explicitly mentioned COVID-19.^32^ While this approach could have been simply a practical advertising strategy during the pandemic, in the Chechen context a direct reference to COVID-19 could have been risky. State authorities’ discourse on COVID-19 was fluctuating between denial and aggressive accusations of spreading the virus. Under such circumstances, avoiding any direct mention of COVID-19 might have been an element of the discursive tactic of being “what the state allows me to be”.

In the narratives concerning their healing practices, grey zone healers often employ the New Age terminology and conceptual framework.^33^ Considering the New Age’s overtly mystical and spiritual connotations, this usage initially appeared counterintuitive. However, it is imperative to emphasise here that in the Soviet and post-Soviet context, the New Age is also associated with science. This fusion arises from the abundance of research activities dedicated to the examination of New Age ideas, such as paranormal phenomena, within the official state-supported scientific system.^34^

As a result, the New Age scientific vocabulary is comfortably used by the grey zone healers, although it requires them to consciously avoid overt connections with the spiritual and occult aspects of the movement. Such lexicon, replete with explanatory mechanisms for various phenomena—such as the evil eye and maleficium (rus. *porcha*)—seems to draw less suspicion due to its dual status as both commonly ubiquitous (terms like “biofield” and “aura” are incorporated into mainstream Russian and Chechen culture), and seemingly grounded in scientific discourse.

Hasan, an unregistered grey zone healer in his 60s operating his own clinic, was asked by Author1 to explain what “evil eye” and “maleficium” are. He responded: “I swear I don't even know. This is a violation of the aura of a person, I think.” Conditions such as evil eye and maleficium are recognized within the tenets of Islam and officially treated in the accredited Islamic Centre, therefore it would appear that discussing them should be a safe endeavour.

Hasan’s discourse, however, leans towards the use of the New Age scientific lexicon. In the course of the conversation, he distanced himself from the domains sanctioned by the state-run Islamic Centre. At the same time, he underscored his proficiency in alleviating the aforementioned conditions through the methods applied in his clinic. He articulates that many individuals seek his services for the purpose of alleviating the “evil eye” or “maleficium,” and reports from his clients indicate marked improvements in their well-being. He elaborates on one of the techniques employed, the “copper box,” asserting that it “reconstructs the aura” and “has properties helping to restore the nervous system, improves blood circulation and memory.”

For grey zone healers, associating themselves with the New Age movement is a tactic similar to asserting their affiliation with biomedical science, although it might appear paradoxical to those unacquainted with the specificity of the Soviet and Russian New Age.^35^

Overall, these healers were faced with an omnipresent state control and surveillance on a daily basis. Using discursive tools, they tried to maintain visible boundaries between their activities and those attributed to “sorcerers”. The latter denomination bore little resemblance to specific, clearly defined practices in the same way the term “Wahhabi” bore only nominal connection to genuine religious practices. Rather, it denoted a constellation of characteristics that could be haphazardly invoked and wielded as an accusation of "terrorist activity," as expounded by Kaliszewska (2020). The healers had to be particularly watchful of the current trends in the state’s discourse about all healing-related issues, and more recently also about the pandemic, where the state’s narrative ranged from denial of its existence to accusations of spreading the disease.^36^

### Navigating the State-Imposed Islamization

The Islamization of the medical landscape, and most notably the formal incorporation of Islamic healing practices in the Islamic Medical Centre in Grozny, is a factor that grey zone healers must consider when narrating their healing methodologies. When discussing Islamic medicine, these healers point to a distinctive facet of the medical landscape that developed in Chechnya in recent years. They draw attention to Islamic stores that offer a variety of medicinal remedies made from herbal raw materials, as well as to practices such as *hijama* (blood cupping) and jinn exorcisms.

While cognizant of the ongoing Islamization of the medical sphere, none of them identifies as an Islamic healer. At the same time, they do not position themselves in an adversarial stance, either. Instead, they discursively manoeuvre between Islamic and non-Islamic medicine. Elmira, for instance, places her therapeutic method, hirudotherapy, on par with *hijama*, yet abstains from categorising herself as a specialist in Islamic medicine. In her words, "Usually, if someone practices Islamic medicine, he also has leeches. Hijama, is translated from Arabic as bloodletting so hirudotherapy is its subvariant, these two types are described in the Quran." Notably, Elmira, for the purpose of self-protection against maleficium (rus. *porcha*) and the evil eye, actively resorts to Islamic medicine herself.

Loma, an apitherapist, says that in Chechnya “they do not pick on me as much […] because treatment with bee products is specified by the Quran and to forbid it, well, you know how it would look.” In his capacity as the proprietor of a multidisciplinary clinic, and as an active participant in national and international honey and beekeeping exhibitions, he feels more confident than herbalists or manual therapists. He deliberately highlights these elements of his practice (such as bee-derived products) that do not fall under the purview of religious authorities’ suspicion.

Sultan, a chiropractor, does not define himself as an Islamic healer, yet he stresses that his ethical approach is directly rooted in faith. He explains, “If you took the money, then you have to take responsibility. We surrender to Allah, and he holds sway over our souls; if we accept [the money], we will have to answer for that money.”

For grey zone healers, as well as for lay people in the Caucasus, the term “Islamic medicine” is also associated with Islamic stores selling a variety of imported goods, including medications and cosmetics labelled as Islamic. The trustworthiness of these Islamic remedies is criticized by most of the grey zone healers for being commercial commodities, and faith in their effectiveness is also diminished because they originate from countries that are often beset by stereotypes within the Russian Federation.

Vezirkhan, a herbalist in his early 60s, voices concerns regarding the impurity and potential side effects of Islamic remedies, noting: “Not clean, from Turkey, from Iran. It's all commercial, I think. I won't even touch them. There are side effects. We have a lot of side effects reported.” In general, healers see Islamic remedies readily available on the market as an unscrupulous means of generating profit. Hasan insists: "Rarely, very rarely you will find people who understand and prescribe Islamic remedies. If you look at the Arab world, places like Egypt, there are charlatans everywhere. Arrogant, uncompromising swindlers; they say that black cumin oil has a shelf life of one year; other oils–two years. Flaxseed oil has a shelf life of three years. This is absurd. Any oil–three months, later it oxidizes…”.

Collectively, grey zone healers employ the tactical ambivalence towards Islamic medicine. They understand the utility of a stance that oscillates between acceptance and suspicion allows them to mitigate suspicion cast upon them. In some cases, religious identity or references to it may therefore be used instrumentally, tactically situating them within a legitimate and state-sanctioned framework. It is pertinent to note that none of the grey zone healers declared themselves as Islamic healers, and the majority of them do not wholeheartedly endorse this genre of healing. At most, they extend partial support, albeit often with considerable reservations.

### Seeking Protection and Demonstrating Professionalism

Each grey zone healer constructs their own medical landscape, predicated upon the resources available to them, personal interests, or established connections. Informal networks, including connections with representatives of the state, are crucial for their activities and adept navigation within these multifarious landscapes. The establishment and cultivation of these networks, frequently necessitating the demonstration of one’s professional competence and utility, are therefore crucial in shaping their perception of security.

While the healers would, to various extents, overtly criticise certain practices and restrictions imposed by the state, they remained acutely aware that their position within the grey zone rendered them inherently vulnerable. In the pursuit of mitigating this vulnerability (which often extended to their families), they established connections that could potentially serve as a safeguard in the event of any antagonistic encounters with state representatives. Such connections are a vital asset for one’s survival in this highly networked society. Thus, the self-assuredness of selected healers relies on their reputation with the authorities; those with a more robust network of connections are better poised to undertake risks or circumvent certain restrictions, offering them an advantage not readily available to their less prominently connected counterparts. This situation bears some resemblance to the circumstance of Salafis/Wahhabis, who, despite their general persecution in Chechnya due to their appearance or other characteristics commonly ascribed to them, may still enjoy relative freedom of expression if they can establish affiliations with the authorities (Raubisko, 2009).

Hasan, a man in his 60s, who opened a multidisciplinary medicine clinic, proudly asserts: "I provided treatment to virtually all of them [representatives of the authorities], including their mothers, their daughters, and their brothers, so to speak.” Other healers mention instances when they provided healing services to an authority figures, or where they have garnered recognition for their practices. Loma boasts that his methods of healing COVID-19 and boosting immunity are known to high-ranked officials, saying: “In Nizhniy Novgorod, there is a German Nikolayevich^37^ who knows all about my methods, and even people in the Parliament [*Duma*] know about it. […] Propolis and beekeeper's wormwood (*Arthemisia absinthium*). I have been treating pneumonia [with them] for fifteen years. (…) Nowadays, many people come to me for tinctures, and even if someone falls ill, they get a milder form of the virus that quickly goes away.”

The discursive tactic demonstrating one’s usefulness and connection to the state apparatus could be aptly termed “*menja nie tronut*” (“they won’t touch me”). It is a phrase connoting a sense of invulnerability, whether consciously or subconsciously wielded, to convey a belief in one's safety within the authoritarian state. This perception of being protected allowed the healers to circumvent certain restrictions. It was particularly visible during the COVID-19 pandemic, especially concerning the use of alcohol, commonly utilised in various healing practices. Alcohol is an ingredient contested by the Chechen state on the grounds of the state-imposed Islamization. It is permissible in halal medicines and cosmetic products, when it does not induce intoxication in the patient, and when there is no alternative for its use (Deuraseh, 2003). The sale of alcohol is very restricted in Chechnya, but due to its historical association with the Russian Federation, it remains legally obtainable. Officially, alcohol can be purchased only between 8.00 am and 10.00 am in the “Lenta” supermarket, and in the “Kupol” restaurant in Grozny City (except in Ramadan). In order to acquire alcohol for their medications (for example, for herbal tinctures), herbalists must purchase it secretly and justify its use in their products.^38^

Yusup, a herbalist, incorporated alcohol in his treatment of pulmonary tract diseases, recounting: “Over the span of 1.5 years, 300 L of alcohol passed through my hands. Previously, I needed 15–20 L a year for tuberculosis patients, or for severe pneumonia, but ever since the onset of COVID, people have begun to use it for prevention…” The increase in the production of the substance, which was in demand in connection with COVID-19, convincingly indicated that the herbalist successfully manoeuvred in the medical market and was able to adapt to new challenges. He was aware of the risks of using alcohol in contemporary Chechnya, but his tactics of dealing with the state authorities allowed him to continue the practice that he started when there was no official ban on alcohol. Loma, an apitherapist, articulated his firm belief in the curative efficacy of alcohol-based tinctures. He was positive that the results of his treatments will shield him from any problems with the authorities.

Overall, the healers employed an approach of showcasing their professionalism, competence, and confidence, with the additional tactic of seeking protection from the patients they cured, especially those holding official positions. The degree to which being well-connected, or even alluding to such connections, could genuinely safeguard them from potential persecution, remains a complex matter to ascertain. This complexity arises from the inherent volatility of loyalties and the reality that one's status can swiftly transition from being perceived as “beneficial and indispensable” to being seen as a threat and an “enemy of the state”.

**Fig. 1:**
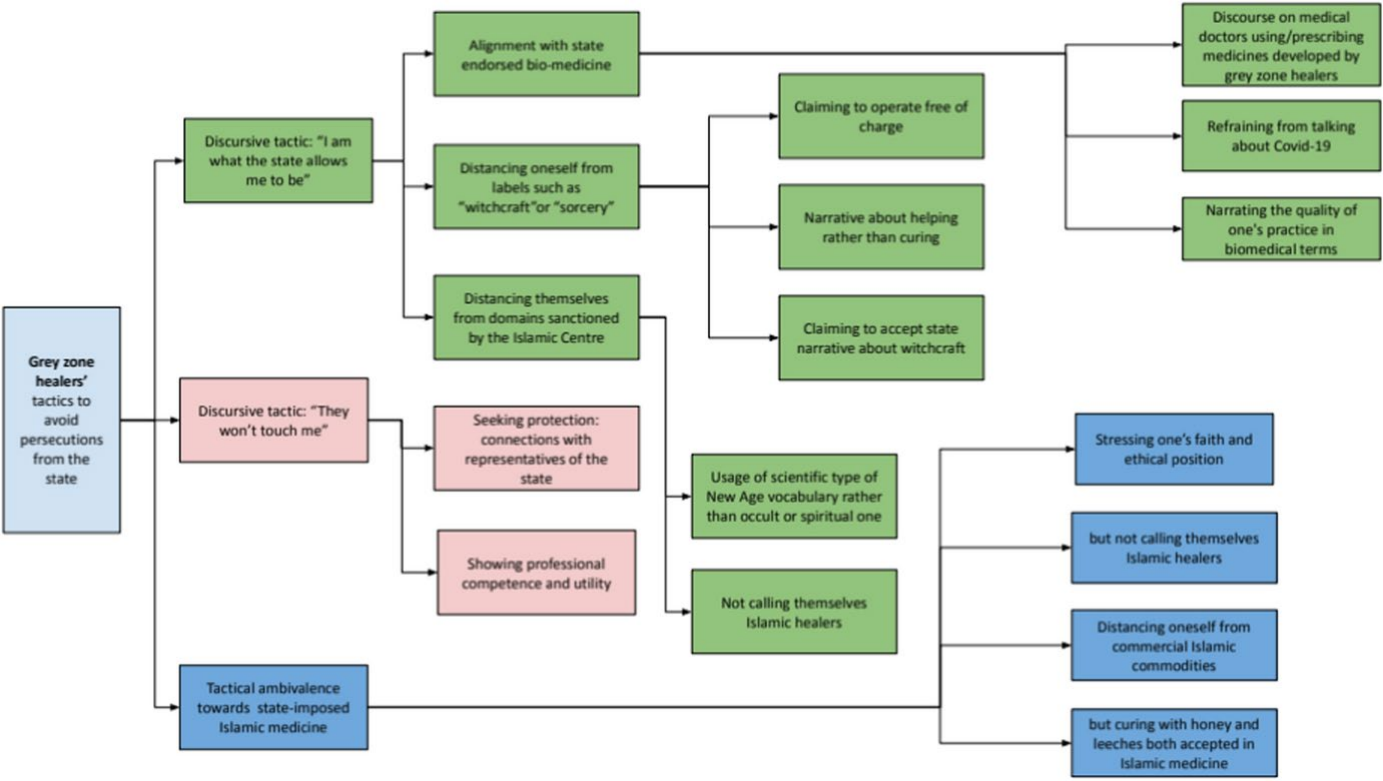
Tactics grey zone healers' employ to avoid persecutions form the state

For the summing up of all of the tactics employed by the grey zone healers, see Fig. 1.

### Study Limitations

As with any research endeavour, particularly one conducted among a vulnerable group in an authoritarian state, our research was burdened with a number of limitations. A notable constraint pertained to the inability to capture audio recordings for every interview, introducing the potential risk of failing to document the precise language and expressions being used. The primary fieldwork was conducted in Russian by a researcher of Russian heritage. While the first author possesses prior experience in researching vulnerable groups, her ethnic background might have inadvertently functioned as a constraining factor, as it is conceivable that certain information could have been withheld from a presumed representative of a colonial power. Furthermore, even though all healers exhibited high proficiency in the Russian language, there remains the possibility that some of their experiences might have been articulated more effectively in their native Chechen tongue. The lack of gender parity among the healers represented another limitation. A further investigation incorporating a comparative analysis encompassing a wider variety of healers, including those affiliated with the Islamic Medical Centre, could provide a more nuanced and comprehensive understanding of how Chechen healers navigate their interactions with the state.

## Conclusions

The discursive tactics employed in response to the state’s control and violence contributed to the adaptability and flexibility exhibited by the healers in the face of unforeseen restrictions that may be imposed upon them. While detachment from sorcery was articulated explicitly, allusions to Islamic practices were less overt, possibly due to a constant flux in the narratives and practices endorsed by the state: what was initially deemed acceptable, could subsequently transform into a prohibited activity.

The process of Islamization, or re-traditionalization of the republic, did not occur in a vacuum. Both the *muftiyat* and the authorities had to respond to the metamorphoses and changing trends of the contemporary world. Consequently, striving to align with the perceived expectations of state authorities necessitated continual adjustments, refinements, and knowledge acquisition. Moreover, healers actively sought to establish personal connections with local authorities, leveraging such relationships as a form of discourse to assert that these affiliations may act as a safeguard in the event of potential conflicts with state officials.

In their attempt to mitigate the risks posed by the state, healers employ a tactical approach to position themselves within what can be termed “the realm of permissible actions.” They achieve this by associating their practices with biomedicine, adopting scientific terminology, and incorporating healing methods characteristic of the Russian New Age movement. In contrast, the alignment with the state-monopolized Islamic medicine is not exhibited as strongly. It is important to mention that medicalization in Chechnya is considerably less forceful than the assertive imposition of Islamic principles.

Differently than in the Western context, in regions where Islam holds a dominant religious position, the medical landscape is distinctly shaped by state authorities, with spiritual healing often assuming a central role (Khadher et al., 2018; Morsy, 1988). In Chechnya, this dynamic resulted in the displacement into the grey zone of healers prescribing treatments focused on the patient’s body. Grey zone healers employ creative tactics that draw upon connotations associated with Islam and tradition to secure their standing within the medical landscape as defined by the Chechen state. It is worth mentioning here that categorizing these healers as either traditional or Islamic was not our objective, as we regard such essentialization as unnecessary.

The tactic mentioned above is used by CAM healers in various cultural contexts. For instance, Ghanaian healers deliberately navigate the boundaries between tradition and modernity (Hampshire & Owusu, 2013). As a result, not identifying themselves as Islamic healers serves as a tactic, enabling them to circumvent judgment in accordance with the standards dictated for Islamic medicine by the state. Those who have this possibility sometimes emphasize components of their therapeutic methodologies that share similarities with Islamic medicine, such as the utilization of bee products and bloodletting. The tactic of discursively dissociating themselves from financial gain allows them to distance themselves from anything that might be associated with fraud, serving as a tool allowing them to evade persecution and association with sorcery. This approach of separating from fraudulent activities is similarly evident in their evaluation of products available for purchase in Islamic shops.

Despite the imposition of numerous COVID-19 restrictions by the state, and the pandemic being a prominent topic in official discourse, most healers did not regard it as an exceptional occurrence. Being accustomed to state violence, they did not substantially alter their performance during the pandemic. The healers were well equipped to address the challenges posed by COVID-19, having been effectively conditioned to manoeuvre within the ever-changing medical landscape of the Chechen Republic. The manner in which the healers dealt with the virus and the pandemic suggests that, for them, COVID-19 was just another obstacle to overcome in their medical milieu inherently permeated by violence and instability.

All but one healer included COVID-19 treatment in their healing practice, although this aspect of their work was not prominently emphasized, and they did not proffer exclusive anti-COVID-19 remedies. They regarded the pandemic as a circumstance to act upon, but they prudently refrained from overtly advertising their activities. They treated COVID-19 by analogy with other known and managed diseases—tuberculosis, pneumonia, and various pulmonary and bronchial ailments (cf. De Meyer and colleagues for the similar practice observed among Belgian Congolese community members (2022)). Although the healers lacked endorsement from biomedicine, the state, or religion, their experiences with COVID-19 demonstrated their capacity to the exigencies of the pandemic and effectively continue their work. Moreover, despite the considerable (and at times oppressive) regulatory intervention by the state in response to the COVID-19 epidemic, the healers ventured into the novel and uncertain market associated with the fight against COVID-19 by offering their therapeutic products.

Lastly, we conclude that it is important to not restrict the research on the reduction of the diversity of medical practices to medicalization, as is often the case in the Western context. While medicalization and the dominance of biomedical practices sanctioned by the state undeniably contribute to structural violence against practitioners operating outside the realm of biomedicine, it is noteworthy that in Chechnya, the global predominance of biomedicine holds relatively limited relevance to alternative healers when juxtaposed with the overriding concerns of state-inflicted violence and the dominion exerted by state-endorsed and state-defined Islamic medicine.

Medical anthropologists rarely discuss the tactics healers employ when facing state violence outside of the biomedical sphere. Their predominant focus has been on patients or healers grappling with biomedical practices enforced by the state. In contrast, political anthropologists reveal day-to-day tactics adopted to negotiate with the state, but may omit practices connected with the medical domain. We therefore believe that our approach provides new insights and encourages scholars to look beyond the dynamics of dominant biomedicine and counter-hegemonic healing practices, shifting attention to other hegemonies beyond the medical sphere.

## Footnotes

1 In non-European/non-Western countries, with Islam as a dominant religion, such as Malaysia or Egypt, spiritual healing is also incorporated into the state-supported system (Khadher et al., 2018; Morsy, 1988).

2 The term CAM encompasses both complementary and alternative medicine. Alternative medicine is usually defined as challenging the commonly accepted medical status quo, or the bureaucratic circumstances of the biomedical health care system (Buda et al., 2006). Complementary medicine is usually defined as one that is more often practised alongside biomedicine rather than in opposition to it (Penkala-Gawęcka, 2017). In this article, we focus on the healers’ status regarding the state regulations rather than biomedicine. Some of the grey zone healers we interviewed can be seen as alternative in regard to Islamic Medical Centre, while the practice of others is considered complementary.

3 All of the healers except Loma—an apitherapy practitioner—did not themselves initiate a conversation with the researcher(s) about COVID-19 and the treatments they could offer for the disease; in no case was COVID-19 treatment presented as their calling card.

4 An entire special issue of this journal concerning COVID-19 titled “COVID-19, Islam and Christianity” was published in April 2021 (JORH, 2021).

5 There are studies of non-Islamic complementary medicine in the region, but, with rare exceptions, they are represented by Russian-language works on historical ethnography (Barnash, 2010; Khasbulatova & Madaeva, 2012; Shavlaeva & Sadulaev, 2020).

6 Field narratives collected in 2010 served as an additional point of reference. Additional interviews or shorter conversations were also conducted in summer 2023 when introducing corrections to the paper.

7 Boris Bolotov [1930], a popular alternative medicine specialist, who developed scientific ideas characteristic for the Soviet New Age. More on the Bolotov ideas and healing methods in English: https://ief-usfeu.ru/en/bezumnye-izobreteniya-borisa-bolotova-merovedenie/; https://kerchtt.ru/en/ostorozhno-lzheuchenyi-boris-bolotov-bezumnye-izobreteniya-borisa/.

8 There is a legitimate question as to the validity of analysing Kadyrov's utilization of Islam within the confines of religious discourse. Although his family had affiliations with the Qadiriyya Sufi order, his public deployment of religious practices derived from Qadiriyya is fundamentally instrumental and imbued with political significance (Raubisko, 2009). We contend that such practices should be approached from a primarily political standpoint, given their instrumental nature. Furthermore, while Qadiriyya Sufi order constitutes a part of broader Sufi networks, in reality there is little international or interregional collaboration.

9 The term “Islamic medicine” lacks a universally established definition. Khadher and colleagues note that some authors writing about Islamic medicine only discuss some aspects of it, meaning that the definition is highly context-dependent (Khadher et al., 2018). In our article, we also adopt a context-sensitive definition of Islamic medicine. We find it crucial, as the state-imposed definition of Islamic medicine shapes the practices of healers in Chechnya. Central to this definition is the concept of disease being largely attributed to the lack of attention paid to the spiritual dimension, and estrangement from the divine will (cf. Salim, 2004). Both physiological and spiritual factors are taken into account in the diagnostic process. In the lay understanding of Islamic medicine, Islamic shops with herbal produce from Arab countries and booklets on the “Prophet’s Medicine” are of crucial importance. These various interpretations of Islamic Medicine are intertwined, but while the state’s perspective is strongly focused on spiritual healing through incantations, in lay definitions herbal treatments play an equally prominent role in the healing process.

10 Umma—the whole community of Muslims bound by the ties of religion.

11 A Spiritual board of Muslims in a given republic; in Chechnya, this body is practically, if not formally, controlled by the state authorities.

12 https://www.kavkaz-uzel.eu/articles/143481/.

13 More on exorcism practices see (Oparin, 2020).

14 https://www.reuters.com/article/uk-russia-chechnya-healers-idUKTRE76K33M20110721.

15 https://www.youtube.com/watch?v=nOLX90Qc7rQ.

16 See for example: https://www.kavkaz-uzel.eu/articles/268913/.

17 In the Caucasus region, it was only the Azerbaijani state representatives who tracked, cautioned, and requested fortunetellers cease practising, but no severe harassment followed https://www.kavkaz-uzel.eu/articles/287692/.

18 Armed groups might have also had other motivations, for example connected with their liberation struggle, but the “witch-hunt” they conducted was not as systemic and was not financed with public money.

19 https://www.youtube.com/watch?v=pWIsYVfKLmY (11:21).

20 More on honour killings (Antonova & Siradzhudinova, 2018).

21 An illustrative instance of this practice in action was the presence of a portrait of Dzerzhynski behind one woman as she underwent public shaming. https://www.uscirf.gov/sites/default/files/2021-10/2021%20Chechnya%20Issue%20Update.pdf.

22 https://www.independent.co.uk/news/world/europe/chechnya-witches-women-halloween-kadyrov-b1949256.htmlhttps://bloknot.ru/obshhestvo/ohota-na-ved-m-v-chechne-politsejskie-zaderzhali-treh-koldunij-i-ustroili-s-nimi-bitvu-e-kstrasensov-831611.html.

23 Among the grey zone healers we spoke to, chiropractors utilising scarves for body measurements considered themselves the most susceptible to accusations of sorcery, because the scarves could be considered fortunetelling paraphernalia.

24 https://www.kavkazr.com/a/elzhurkaev-interview/30211646.html.

25 See for example: https://www.crisisgroup.org/europe-central-asia/caucasus/chechnya-russia/chechnyas-anti-gay-pogrom.

26 On the presence of leeches in ancient medicine, various folk medicines, Islamic medicine, and biomedicine, see for example (Lemke & Vilcinskas, 2020; Vallejo & Gonzalez, 2015).

27 Clinic proprietors Hasan and Zakir, along with Loma, educated in apitherapy, display a heightened sense of confidence and professionalism. They provide elaborate critique of biomedicine while simultaneously attempting to underscore their own biomedical competence. Similarly to the Ghanaian healers (in the Hampshire & Owusu research), they are “ready and eager to collaborate with biomedicine, but as equals. (…) they are not willing to buy into biomedical hegemonies wholesale.” (2013, p. 262).

28 Compare with (Phillips, 2004; Roussou, 2021).

29 Elmira had to pay 40,000 roubles to secure employment in a hospital. Additionally, she receives a lower income than her official salary states, deducting taxes, most likely due to the hierarchical corruption prevalent in the North Caucasus, as documented by Kaliszewska and Schmidt (2022).

30 Official medical doctors and practitioners from the Islamic Centre typically rely on healing as their main or only source of income, eliminating the necessity for any diversion tactics.

31 The use of various sources of income is a common practice in Chechnya, where the opportunities are limited, necessitating individuals to combine multiple sources of income in order to mitigate the risk of sudden financial downturn and to secure a relatively satisfactory revenue. Engagement in healing also serves as a means of accruing supplementary social capital.

32 https://www.instagram.com/p/B-cSj7bIpwi/?igshid=MTc4MmM1YmI2Ng%3D%3D (accessed 23.08.2023).

33 New Age ideas in Russia/USSR in various aspects were influenced by Western New Age ideas, but simultaneously Russian/USSR’s New Age had an important impact on the creation of some Western New Age ideas. Nevertheless there are important differences between them: the influence of intellectuals on the development and transmission of New Age ideas in Imperial Russia and USSR was far more significant than in the West, where it was much more grass-root derived and transmitted by popular culture. The Russian/Soviet intellectuals had many possibilities of a direct personal contact with teachings and traditions of indigenous peoples—in Buryatia, Tibet, the Caucasus and Central Asia; the New Age ideas were prominent among the scientific community, and in various periods were supported by official Soviet science. Feminist ideas and movement were much less vital for Soviet New Age than for the Western one (Menzel, 2012).

34 This type of research would be considered parascience in Western countries (Menzel, 2012).

35 The popularity of parapsychology, both in society and in the scientific community in the 1960s and 1970s, is reflected not only in the fact that Leonid Brezhnev’s sessions with the psychic and healer Evgeniia “Dzhuna” Davitashvili became public knowledge, but also in a bibliography of publications related to paranormal phenomena in specialized and popular scientific books and journals between 1969 and 1981″ (Menzel, 2012, p. 174). Similar level of popularity of such literature is present in the post-Soviet Russian Federation.

36 https://novayagazeta.ru/articles/2020/04/12/84851-smert-ot-koronavirusa-menshee-zlo.

37 German Kolpikov is an apitherapy enthusiast from Nizhny Novgorod.

38 In contemporary Chechnya it is impossible to procure alcoholic products, such as iodine, without a prescription.
