# Correction: Grey Zone Healers and the COVID‑19 Pandemic in Chechnya, Russia

**DOI:** 10.1007/s10943-024-02188-0

**Published:** 2024-11-30

**Authors:** Evgenia Zakharova, Iwa Kołodziejska, Iwona Kaliszewska

**Affiliations:** Faculty of Culture and Arts, Institute of Ethnology and Cultural Anthropology, University of Warsaw, ul. Żurawia 4, 00‑503 Warsaw, Poland

**Correction to: Journal of Religion and Health (2024) 63:4024–4046** 10.1007/s10943-024-02041-4

In this article, the statement in the Funding information section was incorrectly given as ‘National Research Centre (NCN), research grant no. 2020/37/B/HS3/02541’ and should have read ‘National Science Centre, Poland (NCN), research grant no. 2020/37/B/HS3/02541’.

**Incorrect Information**: The study was financed by National Research Centre (NCN), research grant no. 2020/37/B/HS3/02541.

**Correct Information**: The study was financed by National Science Centre, Poland (NCN), research grant no. 2020/37/B/HS3/02541.

